# Establishment of a novel risk score model by comprehensively analyzing the immunogen database of bladder cancer to indicate clinical significance and predict prognosis

**DOI:** 10.18632/aging.103364

**Published:** 2020-06-22

**Authors:** Lingyun Liu, Jinghai Hu, Yu Wang, Tao Sun, Xiang Zhou, Xinyuan Li, Fuzhe Ma

**Affiliations:** 1 Department of Andrology, The First Hospital of Jilin University, Jilin, China; 2 Department of Urology, The First Hospital of Jilin University, Jilin, China; 3 Department of Nephrology, The First Hospital of Jilin University, Jilin, China; 4 Department of Urology, The First Affiliated Hospital of Chongqing Medical University, Chongqing, China

**Keywords:** bladder cancer, immunogen, risk score, prognosis, CACYBP

## Abstract

Background: Bladder cancer (BCa) has the highest incidence of aggressive malignant tumors in the urogenital system and is the ninth most common cancer worldwide. Immune function-related genes (IFRGs), which are plentiful in immune cells and the immune microenvironment (IME), have the potential to assess prognosis and predict the efficacy of immunotherapy. A complete and significant immunogenomic analysis based on abundant BCa genetic samples from The Cancer Genome Atlas (TCGA) will provide insight into the field.

Results: A total of 57 differentially expressed IFRGs were significantly associated with the clinical outcomes of patients with BCa. Functional enrichment analysis showed that these genes actively participated in the KEGG pathway of human cytomegalovirus infection. Based on the IFRGs (CALR, MMP9, PAEP, RBP7, STAT1, CACYBP, ANHAK, RAC3, SLIT2, EDNRA, IGF1, NAMPT, NTF3, PPY, ADRB2 and SH3BP2), the risk scores were calculated to predict survival and reveal the relationships with age, sex, grade, staging, T-stage, N-stage, and M-stage. Interestingly, IFRG-based risk scores (IRRSs) reflected the infiltration of several types of immune cells. The expression of CACYBP was more significant in grade 3, T3 and T4 stages than in earlier grades and T-stages.

Conclusion: Our results highlighted some sIFRGs with remarkable clinical relevance, showed the driving factors of the immune repertoire, and illustrated the significance of IFRG-based individual immune features in the identification, monitoring, and prognosis of patients with BCa.

Methods: Based on the TCGA dataset, we integrated the expression profiles of IFRGs and overall survival (OS) in 430 patients with BCa. Differentially expressed IFRGs and survival-related IFRGs (sIFRGs) were highlighted by calculating the difference algorithm and COX regression analysis in patients with BCa. Based on computational biology, the potential molecular mechanisms and characteristics of these IFRGs were also explored. Using multivariate Cox analysis, new risk scores based on immune-related genes were developed. The expression of CACYBP was verified by qPCR, western blot and immunohistochemistry. The relations between CACYBP and clinical features were proven by immunohistochemistry.

## INTRODUCTION

Bladder cancer (BCa) has the highest incidence of aggressive malignant tumors in the urogenital system and is the ninth most common cancer worldwide [1]. With approximately 165,000 deaths per year worldwide, it has become the 13^th^ cause of death from cancer [2]. Most likely caused by the differences in risk factors, detection and diagnostic practices, and the availability of treatments, the geographical variations in incidence and mortality are noticeable, with the highest morbidity in North America and Europe and the greater mortality in developing regions [3]. Additionally, significant medical expenditures might be due to the high recurrence rates and advanced presenting stage, limiting the survival benefit from various treatments. Therefore, forecasting the progression and prognosis of BCa through several novel and sensitive biomarkers might reduce the proportion of patients with aggressive BCa as well as guide a more appropriate therapeutic schedule.

Consisting of immune cells and stromal cells together with extracellular vesicles and other molecules, the tumor microenvironment (TME), as a crucial regulator of gene expression, has been demonstrated to be closely involved in oncogenesis, development and therapeutic processes [4–7]. Additionally, some studies have demonstrated that a series of immune function-related genes (IFRGs) plentiful in immune cells and the immune microenvironment (IME) have the potential to assess and predict the sensitivity and efficacy of immunotherapy; however, the value of IFRGs in the IME for therapy and prognosis estimations of BCa remain problematic.

Beginning with the favorable response of bacillus Calmette–Guérin (BCG) intravesical instillation for BCa reported in 1976, the door of immunotherapy for BCa was gradually opened [8]. To date, BCG, together with pirarubicin and other chemotherapeutics, has been regarded as the standard adjuvant treatment for nonmuscle invasive bladder cancer (NMIBC), which accounts for approximately 70% of BCa [9, 10]. Although the therapeutic effect of BCG in NMIBC is significant and the tumor-specific immunity induced by BCG has been extensively studied, the mechanisms remain unclear owing to the multiple biological processes involved, including congenital immunity and acquired immunity.

In addition to NMIBC, although radical cystectomy and chemotherapy remain the standard therapies for muscle invasive cancer (MIBC), the alarming mortality of advanced BCa prompts urologists to discover more appropriate and personalized therapeutic schedules. Recently, the identification of immune checkpoints (PD-1/PD-L1) and chimeric antigen receptor T-cells (CAR-T) began raising the profile of immunotherapy in MIBC and metastatic cancer [11]. Powles demonstrated the satisfying efficacy and safety of atezolizumab (the first anti-PD-1/PD-L1 drug investigated in BCa) for patients with metastatic BCa following chemotherapy [12]. Since then, durvalumab, avelumab and a series of other anti-PD-1/PD-L1 drugs have been approved by the FDA as second-line therapies for advanced/metastatic BCa [13]. However, less than half of advanced/metastatic BCa is sensitive to anti-PD-1/PD-L1 drugs; thus, screening and detecting some immunotherapy response predictors and prognostic estimation biomarkers is worthwhile.

Therefore, we designed the research to provide insight into the clinical potency of the IME and IFRGs on BCa prognosis estimation and promising therapeutic prediction for immunotherapy. We extracted a series of IFRGs in the IME predicting poor prognosis in patients with BCa, and together with tumor-related information, we further computed their connections with overall survival (OS). The results shed light on clarifying the approaches and underlying mechanisms of IFRGs in BCa and establish a more personalized precision prediction model for immunotherapy.

## RESULTS

### IFRGs with differential expression

**Figure 1:**
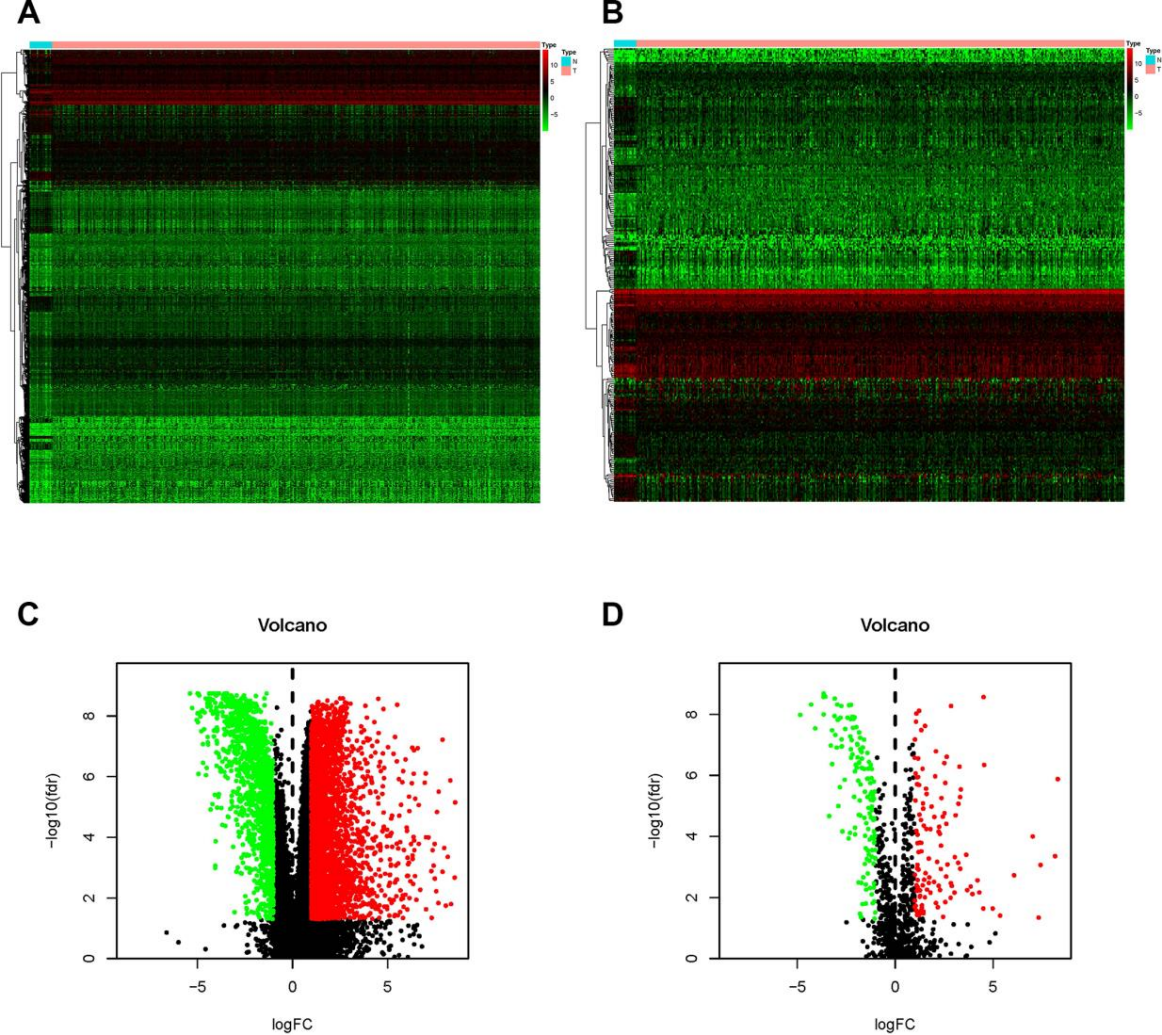
**Differentially expressed BCa genes and IFRGs.** Heatmap (**A**) and volcano plot (**C**) show the differentially expressed genes between BCa tissues and nontumor tissues. The black dots represent genes without differential expression; the red dots represent the significantly upregulated genes, and the green dots represent downregulated genes. FDR < 0.05, log_2_ | FC | >1 and P < 0.05. Immune function-related genes (IFRGs) with various expression levels are illustrated in the heatmap (**B**) and volcano plot (**D**). The black dots represent IFRGs without differential expression; the red dots represent upregulated IFRGs, and the green dots represent downregulated IFRGs. FDR < 0.05, log_2_ | FC | >1 and P < 0.05.

**Figure 2:**
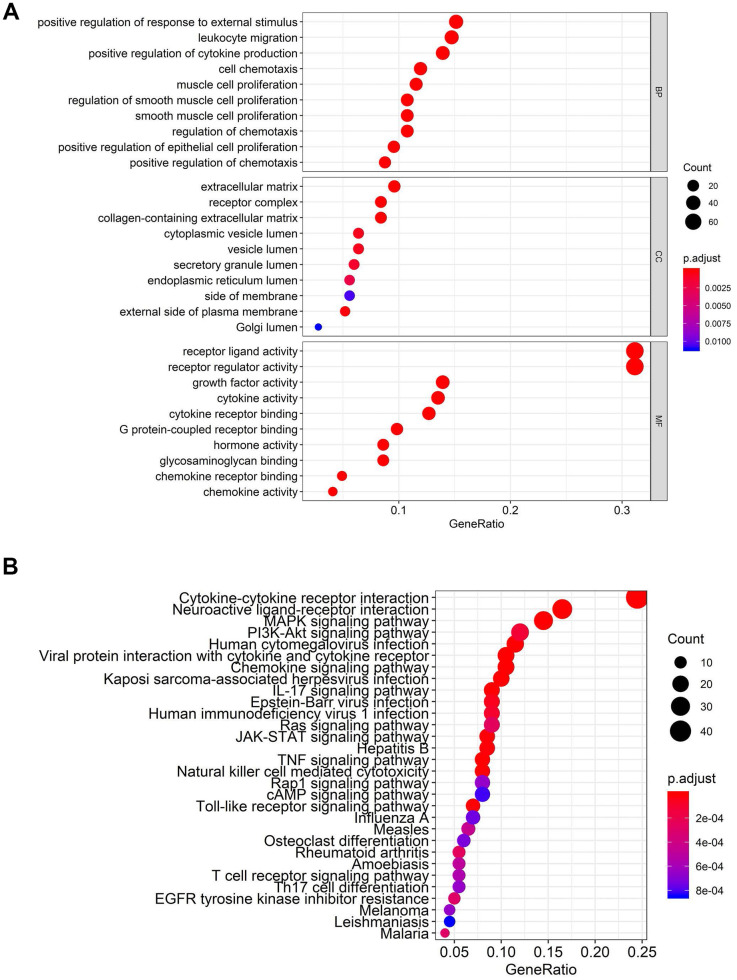
**Functional enrichment analysis of differentially expressed IFRGs.** The top pathways of IFRGs are shown in biological process, cellular component, molecular function (**A**), and KEGG pathway (**B**).

According to the Limma algorithm, we first recognized 4881 differentially expressed BCa genes, of which 1423 were downregulated and 3458 were upregulated (Figure 1A and 1C). To investigate the immune correlations of these genes, we further confirmed 260 IFRGs, including 140 downregulated and 120 upregulated IFRGs (Figure 1B and 1D). We next discovered the functional enrichment of these identified IFRGs by gene ontology (GO). Interestingly, “receptor ligand activity”, “positive regulation of response to external stimulus” and “extracellular matrix” were the most common entries among molecular functions (MF), biological processes (BP) and cellular components (CC), respectively (Figure 2A). Meanwhile, we found, based on Kyoto Encyclopedia of Genes and Genomes (KEGG) pathways, that “cytokine-cytokine receptor interaction” was the most frequently enriched pathway of these IFRGs (Figure 2B). According to the GO and KEGG analysis results, extracellular components are probably the potential terms triggering cell-cell interactions, which inspired us to focus more attention on the elements and requirements of achieving intercellular communication, such as receptors, ligands and extracellular signals, in further studies of BCa.

### The characteristics of sIFRGs

**Figure 3:**
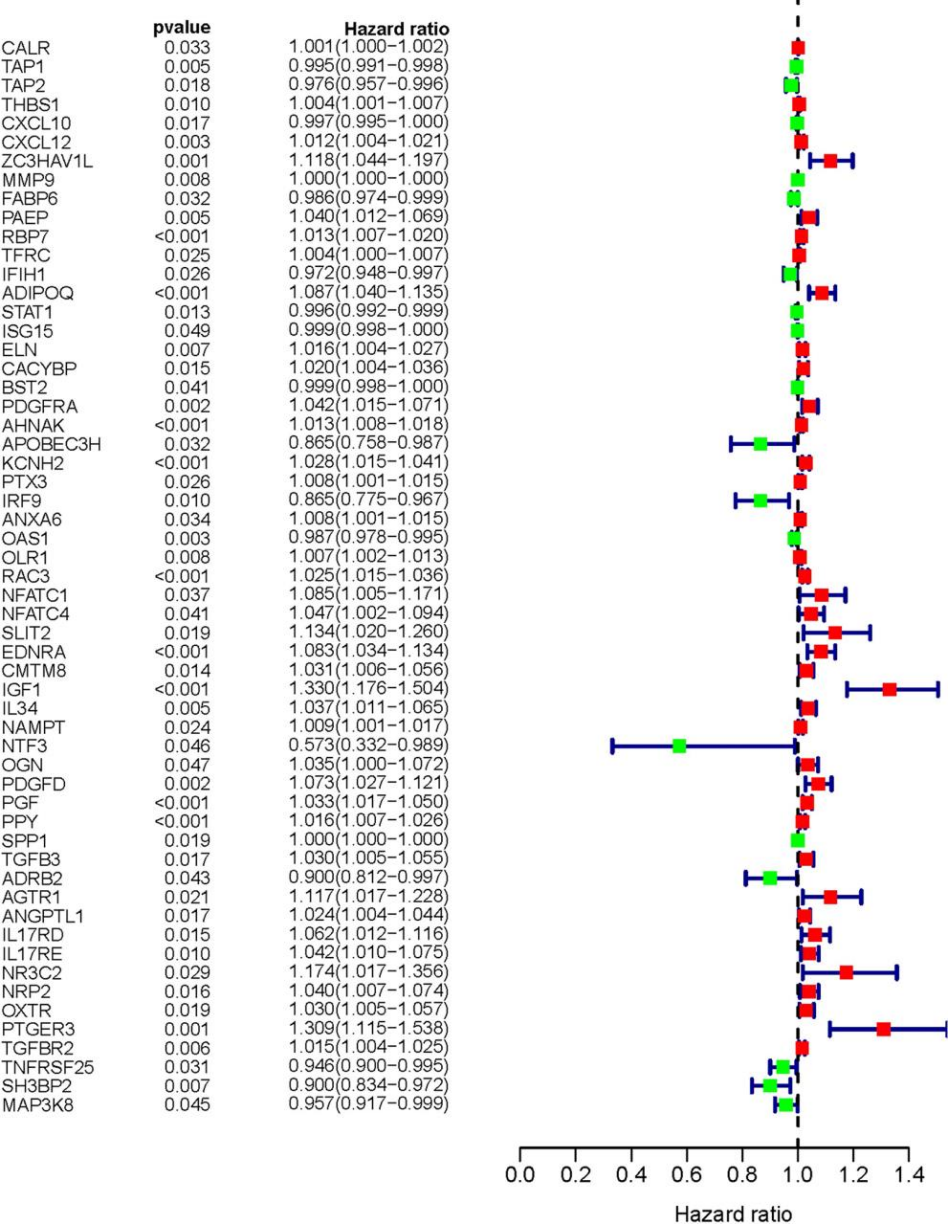
**Survival-related values of sIFRGs.** Forest plot of the hazard ratios showing the survival-related values of sIFRGs. Red parts represent upregulated sIFRGs, and green parts represent downregulated sIFRGs.

With the consideration of predicting prognosis, we explored valid IFRGs as biomarkers to guide clinical treatments. Together with clinical outcomes, we analyzed the survival correlation of these IFRGs, of which 57 were closely involved in the progression of BCa and significantly related to OS (sIFRGs) (P<0.05). To investigate the potency of predicting prognosis and the characteristics of BCa, a forest plot of the 57 sIFRGs was employed to detect the expression profiles in non-BCa and BCa samples (Figure 3 and Table 1). As illustrated in the forest plot, the majority of these genes were upregulated in BCa samples (Figure 3). Consistently, the hazard ratio forest plot revealed that the largest number of sIFRGs played negative roles. The aforementioned results suggest that these upregulated sIFRGs corelating with poor prognosis seem to be the underlying genetic markers.

**Table 1:** Survival-related IFRGs.

**Gene**	**HR**	**HR 95% low**	**HR 95% high**	**P value**
CALR	1.000847	1.000068	1.001627	0.033166
TAP1	0.994957	0.991442	0.998484	0.00511
TAP2	0.97614	0.956832	0.995838	0.017828
THBS1	1.003997	1.000965	1.007037	0.009737
CXCL10	0.997413	0.99529	0.99954	0.017183
CXCL12	1.01237	1.0042	1.020606	0.002941
ZC3HAV1L	1.117514	1.043556	1.196714	0.001471
MMP9	1.000238	1.000062	1.000413	0.007935
FABP6	0.986137	0.973633	0.9988	0.032006
PAEP	1.039889	1.011963	1.068585	0.00486
RBP7	1.01316	1.006577	1.019786	8.46E-05
TFRC	1.003791	1.000486	1.007108	0.024532
IFIH1	0.972156	0.948279	0.996634	0.026031
ADIPOQ	1.086549	1.040255	1.134904	0.000187
STAT1	0.995618	0.992191	0.999056	0.012528
ISG15	0.999189	0.998382	0.999997	0.049192
ELN	1.015619	1.004184	1.027185	0.007304
CACYBP	1.020015	1.003847	1.036444	0.01506
BST2	0.998999	0.998039	0.999959	0.040922
PDGFRA	1.042349	1.014778	1.070668	0.002425
AHNAK	1.013151	1.008457	1.017866	3.50E-08
APOBEC3H	0.8651	0.75789	0.987475	0.031819
KCNH2	1.027957	1.015028	1.041051	1.96E-05
PTX3	1.007881	1.000928	1.014882	0.026235
IRF9	0.865436	0.774896	0.966554	0.010368
ANXA6	1.007771	1.000571	1.015022	0.034337
OAS1	0.986883	0.978441	0.995397	0.002589
OLR1	1.007291	1.001873	1.012737	0.008286
RAC3	1.025256	1.01461	1.036013	2.82E-06
NFATC1	1.084623	1.005009	1.170544	0.03676
NFATC4	1.047038	1.001776	1.094346	0.041484
SLIT2	1.133908	1.020462	1.259965	0.019461
EDNRA	1.083124	1.034283	1.134271	0.000694
CMTM8	1.030716	1.006205	1.055824	0.01375
IGF1	1.329928	1.176249	1.503687	5.34E-06
IL34	1.037458	1.010888	1.064727	0.005469
NAMPT	1.009155	1.001195	1.017179	0.024101
NTF3	0.573343	0.332298	0.989239	0.045626
OGN	1.035453	1.000478	1.071652	0.0469
PDGFD	1.07313	1.027289	1.121016	0.001531
PGF	1.033276	1.016941	1.049873	5.67E-05
PPY	1.016415	1.007358	1.025554	0.000364
SPP1	1.000133	1.000022	1.000244	0.018935
TGFB3	1.030033	1.005408	1.055262	0.016536
ADRB2	0.899691	0.812115	0.99671	0.04307
AGTR1	1.117288	1.01665	1.227888	0.021288
ANGPTL1	1.02378	1.004182	1.043759	0.017162
IL17RD	1.062429	1.011875	1.115509	0.01491
IL17RE	1.041872	1.009706	1.075063	0.010359
NR3C2	1.174191	1.016974	1.355711	0.028563
NRP2	1.040162	1.007408	1.073982	0.015863
OXTR	1.030436	1.004916	1.056605	0.019119
PTGER3	1.309258	1.114799	1.537637	0.001021
TGFBR2	1.014635	1.004206	1.025172	0.005848
TNFRSF25	0.946165	0.899665	0.995069	0.031381
SH3BP2	0.900158	0.833929	0.971647	0.006983
MAP3K8	0.956903	0.916625	0.998951	0.044665

Note: HR: Hazard Ratio.

**Figure 4:**
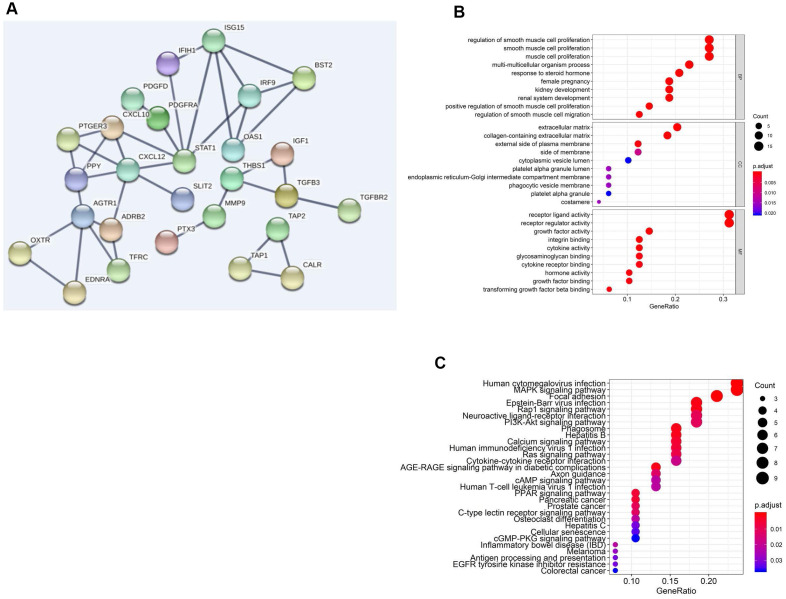
**Functional enrichment analysis of differentially expressed sIFRGs.** PPI network (**A**) of sIFRGs and the top pathways of sIFRGs are shown in biological process, cellular component, molecular function (**B**), and KEGG pathway (**C**).

Through protein-protein interaction (PPI) network analysis, we further found that AGTR1, STAT1, ISG15 and CXCL12 were the core genes among the 57 sIFRGs (Figure 4A). Furthermore, the functional enrichment analysis of the 57 sIFRGs revealed similar results to all 260 IFRGs; for example, “receptor ligand activity”, “regulation of smooth muscle cell proliferation” and “extracellular matrix” were the most enriched terms in MF, BP and CC, respectively (Figure 4B). Unlike the KEGG analysis of the 260 IFRGs, “human cytomegalovirus infection” was identified to be the most enriched among the pathways of sIFRGs, and “cytokine-cytokine receptor interaction” and “neuroactive ligand-receptor interaction” were also enriched in the KEGG analysis of the 57 sIFRGs (Figure 4C), which indicates the significant role of intercellular activity once again.

### TF-related network

**Figure 5:**
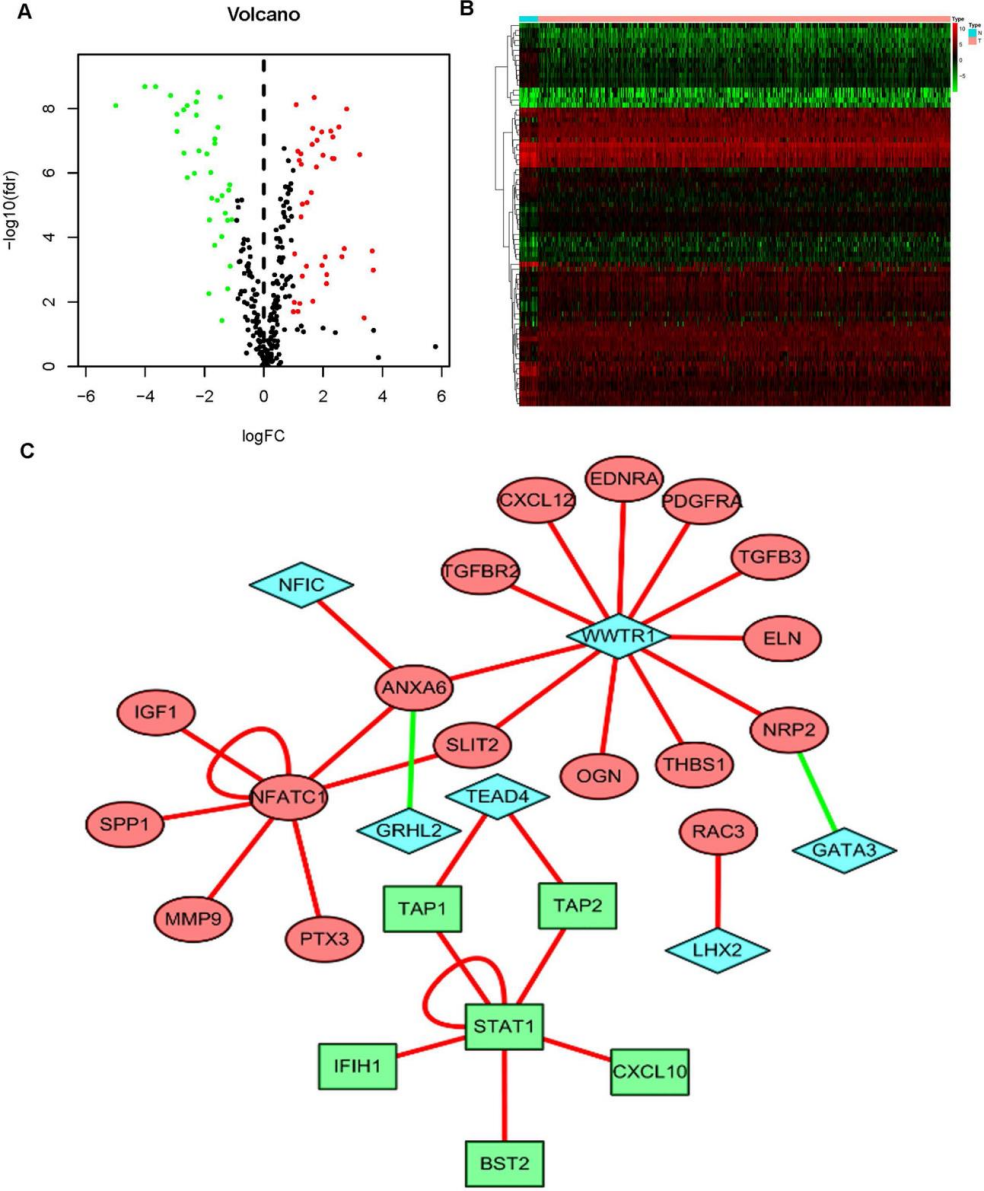
**The mediated relationships between sIFRGs and differentially expressed transcription factors.** Volcano plot (**A**) and heatmap (**B**) showing the differentially expressed transcription factors (TFs). The black dots represent TFs without differential expression; the red dots represent upregulated TFs, and the green dots represent downregulated TFs. FDR < 0.05, log_2_ | FC | >1 and P < 0.05. The regulatory network (**C**) was composed of relevant TFs and sIFRGs. The red circles represent upregulated sIFRGs, the green rectangles represent downregulated sIFRGs, and the blue rhombuses represent relevant TFs. The red lines represent positive regulation, and the green lines represent negative regulation.

To further detect the underlying mechanisms involved in the clinical differences caused by these sIFRGs, we attempted to discover their regulatory mechanisms. Eventually, 318 transcription factors (TFs) were examined, and we found 77 TFs with differential expression, of which 41 TFs were upregulated and another 37 TFs were downregulated (Figure 5A and 5B). According to the relevant TFs (Supplementary Table 1) and 57 sIFRGs, we then implemented a regulatory network illustrating the relationships among these sIFRGs (Figure 5C). A series of TFs were found to be related to sIFRGs, of which “STAT1” and “NFATC1” showed a closer correlation with their corresponding sIFRGs. The results inspired us to further explore whether these TFs are truly regulated by sIFRGs and whether the variations in TFs play significant roles in the progression and prognosis of BCa in future studies.

### Clinical consequences assessment

**Figure 6:**
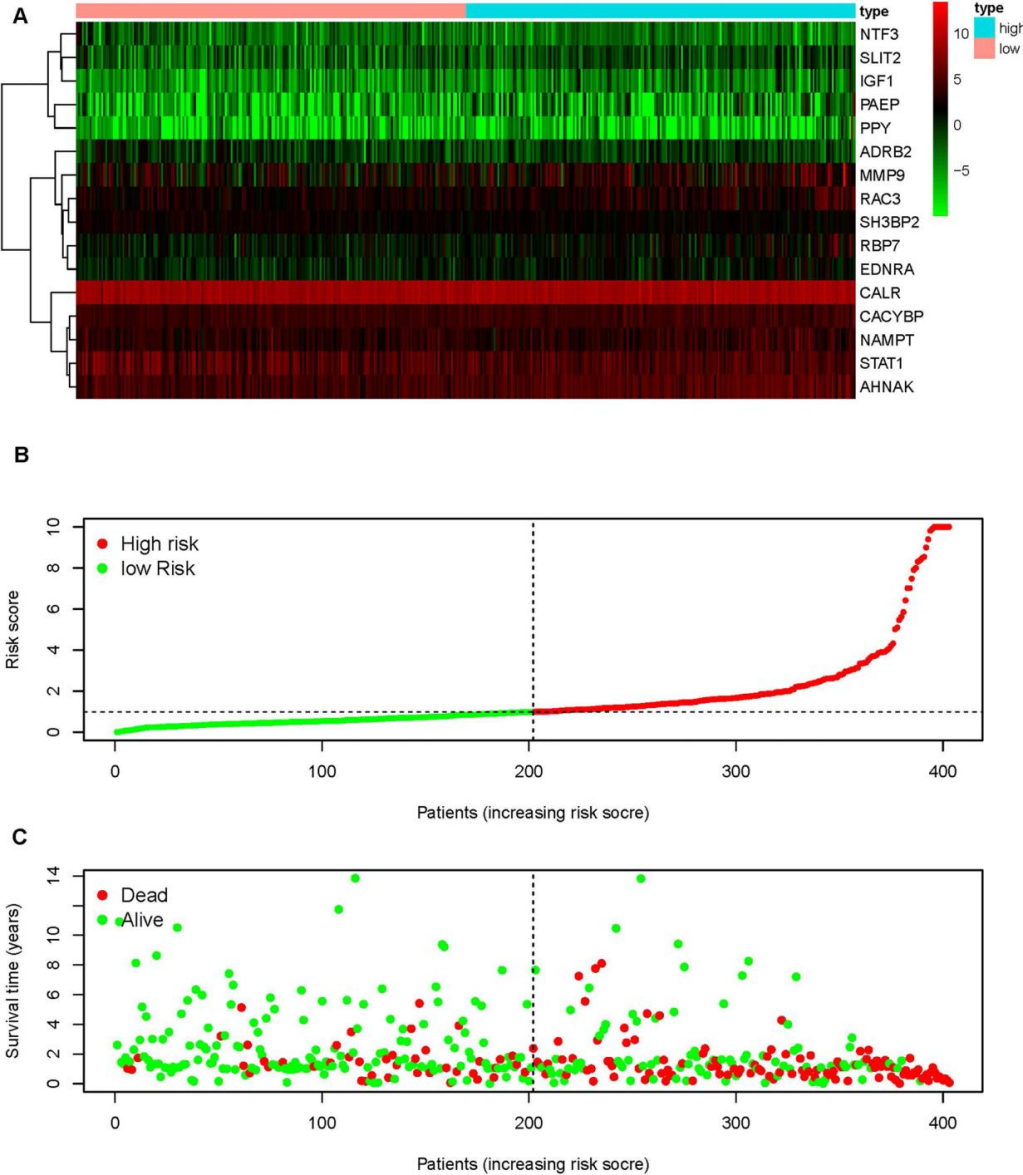
**An immunogen-related risk score model (IRRS) was established according to sIFRGs.** Heatmap of expression profiles of contained sIFRGs (**A**). The red parts represent upregulation, the green parts represent downregulation, and the black parts represent no difference. FDR < 0.05, log_2_ | FC | >1 and P < 0.05. Distribution of groups and risk score rank (**B**). Survival status between the low-risk group and the high-risk group (**C**).

**Figure 7:**
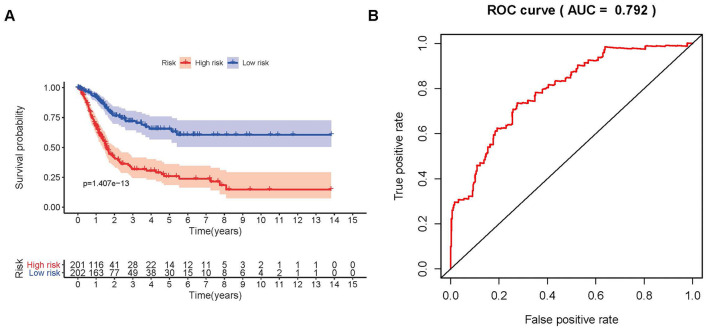
**Survival curve and receiver operating characteristic (ROC) curve.** The survival curve between the low-risk group and the high-risk group (**A**). Survival-related receiver operating characteristic (ROC) curve validation of the survival value of the risk score (**B**).

To distinguish the heterogeneous clinical prognostic outcomes, based on the differential expression of sIFRGs, we performed a risk scoring model to divide patients with BCa into a high-risk group and a low-risk group. (Figure 6A–6C). The formula was as follows: [Expression level of CALR * (0.000964)] + [Expression level of MMP9 * (0.000387)] + [Expression level of PAEP* (0.035026)] + [Expression level of RBP7 *(0.014213)] + [Expression level of STAT1 * (-0.00786)] +[Expression level of CACYBP * (0.019418)+[Expression level of AHNAK * (0.016702)]+[Expression level of RAC3 * (0.015394)]+[Expression level of SLIT2* (-0.20438)]+[Expression level of EDNRA * (0.098941)]+[Expression level of IGF1 * (0.264303)]+[Expression level of NAMPT * (0.013366)]+ [Expression level of NTF3 * (-0.66003)]+[Expression level of PPY * (0.014214)]+[Expression level of ADRB2 * (-0.13911)]+[Expression level of SH3BP2 * (-0.0922)]. Based on the divisions of the IRRS, we further detected the survival probabilities of high-risk and low-risk patients with BCa. As illustrated in Figure 7A, low-risk BCa patients obtained higher survival probabilities than high-risk patients, which indicated that the IRRS could be regarded as a significant tool for the potential outcomes of patients with BCa. The area under the ROC curve was 0.792, suggesting moderate potential for the prognostic signature based on IRGs in survival monitoring (Figure 7B). Univariate Cox regression analysis indicated that the risk score, T-stage and N-stage were closely related to OS (p<0.05) (Table 2). In the multivariate Cox regression analysis, only the risk score was ascertained to have potential for predicting OS (Table 3). Therefore, we think it will be reasonable to believe that the “risk score”, which is both significant in univariate and multivariate Cox regression analysis, is promising for use in predicting the prognosis of BCa.

**Table 2:** Univariate and multivariate analysis of bladder cancer.

**Variables**	**Univariate analysis**				**Multivariate analysis**			
	**HR**	**HR 95% low**	**HR 95% high**	**P value**	**HR**	**HR 95% low**	**HR 95% high**	**P value**
**Age**	1.026750146	0.996811959	1.057587494	0.080382378	1.022499415	0.992503387	1.053402	0.143021228
**Gender**	0.6036572	0.332997546	1.09430841	0.096306254	0.639685652	0.363043374	1.127131808	0.122134081
**Stage**	1.913027307	1.291618442	2.833401381	0.001208593	1.199227402	0.60675845	2.370212335	0.601218975
**T-stage**	1.721152973	1.13007	2.621401822	0.011418547	1.242209542	0.739265571	2.087320994	0.412734429
**M-stage**	1.909066632	0.590116246	6.175961818	0.280382405	1.199837781	0.386899185	3.720893593	0.752380095
**N-stage**	1.501234122	1.114049602	2.022983434	0.007593787	1.143655033	0.682825713	1.915491477	0.609977182
**Risk score**	1.462695822	1.298618364	1.647504091	3.74E-10	1.123890935	1.069330563	1.181235136	4.22E-06

Note: HR: Hazard Ratio.

**Table 3:** The relationships between the compositions of the risk scores and the tumor characteristics.

**Genes**	**Age (≥65/<65)**	**Gender (male/female)**	**Grade (high/low)**	**Stage (III-IV/I-II)**	**T-stage (T3-4/T1-2)**	**M-stage (M1/M0)**	**N-stage (N1-3/N0)**
	**t (P)**	**t (P)**	**t (P)**	**t (P)**	**t (P)**	**t (P)**	**t (P)**
CALR	-0.264(0.792)	0.455(0.651)	4.913(7.177E-05)	-1.414(0.161)	-1.128(0.262)	2.432(0.047)	3.169(0.366)
MMP9	0.078(0.938)	0.658(0.513)	5.359(3.282E-07)	-1.636(0.105)	-1.991(0.048)	-1.089(0.322)	9.053(0.029)
PAEP	-0.948(0.345)	0.128(0.899)	2.01(0.046)	-1.686(0.095)	-1.804(0.074)	-0.141(0.891)	3.774(0.287)
RBP7	-0.697(0.487)	1.141(0.262)	2.232(0.027)	-2.058(0.042)	-1.864(0.065)	-1.316(0.245)	7.409(0.060)
STAT1	0.2(0.842)	-0.067(0.947)	6.412(1.267E-07)	-2.099(0.039)	-2.18(0.031)	2.94(0.017)	1.541(0.673)
CACYBP	-1.315(0.191)	-0.867(0.390)	5.58(4.623E-06)	-2.2(0.030)	-2.138(0.035)	-1.736(0.142)	8.478(0.037)
AHNAK	-0.76(0.449)	1.447(0.157)	5.62(7.216E-07)	-3.7(3.095E-04)	-3.933(1.288E-04)	0.942(0.382)	10.444(0.015)
RAC3	-0.382(0.703)	1.04(0.305)	2.793(0.007)	-0.544(0.588)	0.066(0.947)	-1.129(0.309)	5.199(0.158)
SLIT2	-1.126(0.262)	1.062(0.295)	3.459(0.001)	-3.562(5.009E-04)	-3.562(4.954E-04)	-1.72(0.145)	9.025(0.029)
EDNRA	-2.084(0.039)	0.62(0.538)	5.466(1.396E-06)	-4.285(3.285E-05)	-4.398(2.098E-05)	-1.642(0.158)	7.209(0.066)
IGF1	0.094(0.925)	0.856(0.397)	2.933(0.004)	-3.557(5.508E-04)	-3.111(0.002)	-0.965(0.373)	8.096(0.044)
NAMPT	-0.149(0.882)	1.157(0.254)	4.022(2.108E-04)	-3.065(0.003)	-2.533(0.012)	0.822(0.440)	5.068(0.167)
NTF3	-0.795(0.428)	-0.27(0.787)	-0.361(0.722)	-0.075(0.940)	0.091(0.927)	-0.756(0.480)	1.695(0.638)
PPY	-1.26(0.210)	-1.85(0.066)	2.832(0.005)	-1.702(0.091)	-1.891(0.061)	-0.7(0.513)	2.971(0.396)
ADRB2	0.777(0.439)	-0.956(0.341)	1.075(0.288)	-0.625(0.533)	-0.611(0.542)	0.318(0.762)	3.084(0.379)
SH3BP2	0.798(0.427)	0.214(0.832)	0(1.000)	1.799(0.077)	0.9(0.371)	-0.121(0.908)	9.449(0.024)
Risk score	-1.485(0.140)	2.511(0.017)	6.993(8.874E-11)	-5.847(4.211E-08)	-5.452(2.772E-07)	-1.387(0.223)	19.684(1.974E-04)

Note: t: t value of student's t test; P: P-value of student's t test.

### Clinical use of the risk score signature

**Figure 8:**
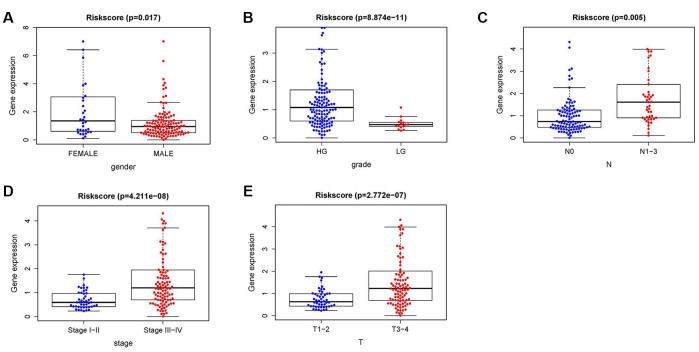
**The relationships between the IRRS and different clinical features.** Relationships between the IRRS and sex (**A**), tumor grade (**B**), N-stage (**C**), tumor staging (**D**) and T-stage (**E**).

To further investigate the relevance of the sIFRGs and clinicopathological features of BCa, we analyzed the relationship between the IRRS and clinical and demographic characteristics, including age, sex, grade, staging, T-stage, N-stage, and M-stage. Under the IRRS, the scores of female patients (Figure 8A), high-grade patients (Figure 8B), patients with advanced N-stage (Figure 8C), advanced staging (Figure 8D) and advanced T-stage (Figure 8E) significantly increased. However, no differences were observed with respect to age or in terms of M-stage. The above results elucidate some clinicopathological and demographic characteristics that are sensitive to the IRRS and further corroborate the clinical application value of the model.

We also analyzed the relationships between the compositions of the risk scores and the aforementioned tumor characteristics. As illustrated in Supplementary Figure 1, some sIFRGs, including the higher expression of AHNAK and CACYBP, were closely correlated with advanced grade, staging, T-stage and N-stage. Meanwhile, strong positive relevance was observed between EDNRA expression and age, grade, stage and T-stage. Furthermore, the increased expression of IGF1 obviously positively influenced grade, staging and T-stage. The lower expression of MMP9 showed a negative correlation with grade and T-stage. The expression of NAMPT and SLIT2 was positively related to grade, staging and T-stage. STAT1 is a remarkable IFRG that is differentially expressed in various grades, stages, T-stages and M-stages. The high expression of CALR was correlated with advanced grade and early M-stage. The overexpression of RBP7 has a stronger relationship with more advanced staging and grade. The increased expression of PAEP, PPY and RAC3 was proven to be closely correlated with a higher grade. SH3BP2 overexpression indicated the early N-stage. Detection of the roles of different sIFRGs in indicating tumor characteristics provides insight into the further discovery of biomarkers.

**Figure 9:**
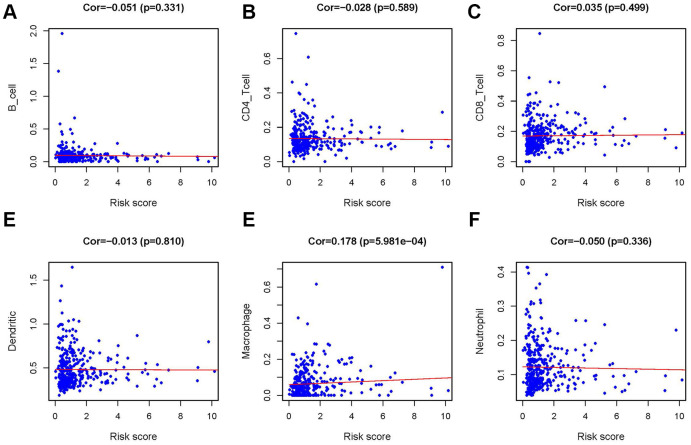
**Relationship between the IRRS and infiltration abundances of six types of immune cells.** The relationships were examined using Pearson correlation analysis. B cells (**A**); CD4 T cells (**B**); CD8 T cells (**C**); dendritic cells (**D**); macrophages (**E**); neutrophils (**F**).

In order to verify whether the immune genome accurately reflects the state of the tumor’s immune microenvironment, we analyzed the relationships between sIFRGs and immune cell infiltration (Figure 9A–9F). We found that macrophages showed the most significant relationship with sIFRGs (Figure 9E), which is probably due to the remarkable effects of macrophage polarization on the tumorigenesis, progression, prognosis and tumor behaviors of BCa. The results motivated us to further discover the underlying functions and mechanisms in future studies.

### CACYBP was overexpressed in patients with BCa, especially with advanced grades and T-stages

**Figure 10:**
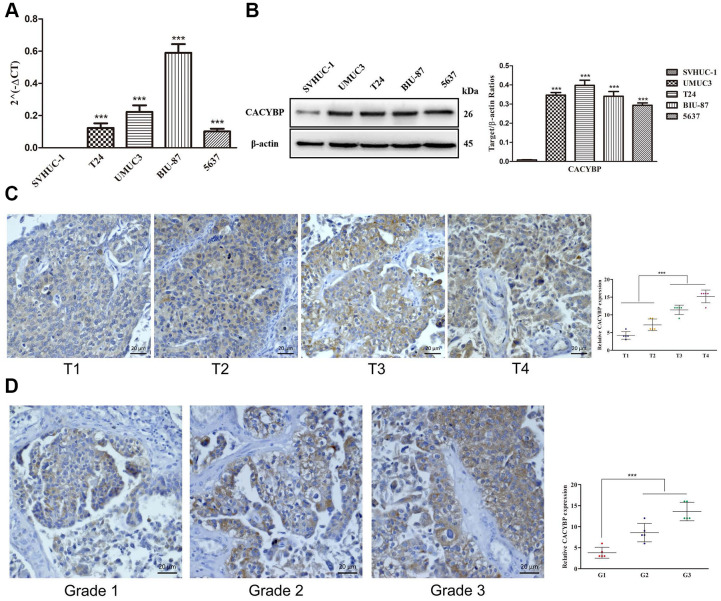
**The overexpression of CACYBP in patients with BCa and its correlation with grades and T-stages.** The results of RT-qPCR of CACYBP expression in bladder cancer cells and normal urothelial cells (**A**). Western blot analysis showed that the expression of CACYBP was higher in BCa cell lines than in normal urothelial cells (**B**). Increasing expression levels of CACYBP were detected by immunohistochemistry (**C**). CACYBP was also increased in various grades incrementally (**D**).

To further verify the relationships between sIFRGs and the clinicopathologic features, as well as discover the roles of sIFRGs in indicating clinical prognosis, CACYBP was selected. Consistently, the correlations of CACYBP expression levels and clinicopathological features of BCa as well as OS were similar to those of the integrated sIFRGs (Supplementary Figure 2). We further detected the expression levels of CACYBP in bladder cancer tissues and normal adjacent tissues of 50 patients with various grades, stages and T-stages *in vivo*. In addition, the expression of CACYBP at the genetic and protein levels was analyzed among normal urothelial cells and various BCa cell lines. As illustrated in Figure 10B, compared with the urothelial cell line SVHUC-1, the higher expression of CACYPB was examined in BCa cell lines including T24, UMUC3, BIU-87 and 5637 at the protein level. Accordingly, consistent with the western blot results, qRT-PCR demonstrated higher expression in BCa cell lines at the genetic level (Figure 10A). Next, we utilized pathological tissue slices to detect CACYPB expression *in vivo*. We found that the expression of CACYBP was more significant in T3 and 4 stages, grade 3 compared with that in earlier T-stages (Figure 10C) and grades (Figure 10D). The aforementioned results suggest that CACYBP, one of the sIFRGs, has the potential to indicate the clinical prognosis of BCa.

## DISCUSSION

Conventionally, surgery and chemotherapy are regarded as the standard managements for BCa, especially for MIBC [1]. However, recent studies revealed that approximately 50% of patients with advanced and metastatic BCa are ineligible and insensitive to standard strategies with cisplatin chemotherapy; thus, the discovery of more personalized precision medicines is eagerly anticipated [14]. With a better understanding of CAR-T cells, immune checkpoints and the satisfactory performance of checkpoint blockage, immunotherapy has ascended to the high-profile treatment strategy in the area of cancer research, while increasing numbers of anti-PD-1/PD-L1 drugs, including pembrolizumab and atezolizumab, are successively approved and display clinical efficacy [15–17]. Notably, the treatment response rate has been proven to be closely connected with the expression level of PD-1/PD-L1 and other immune-related biomarkers, and less than half of patients with MIBC show pleasing outcomes following checkpoint blockage [18–20]. Therefore, discovering significant IFRGs that have the potential to predict and assess treatment prospects is of great interest.

Although the characteristics and significance of IFRGs on tumor invasion, metastasis, progression and immune-related responses have been well established in a proportion of cancers, genome-wide and complete analyses revealing the mechanisms of IFRGs on BCa remain confusing and need to be fully explored [21–23]. During this analysis process, large numbers of patients with BCa were included in the results, which further improved the persuasion in providing clinical evidence. In addition, some specific IFRGs showing obvious differences in variable risk factors have been demonstrated to participate in the progression and prognosis of BCa. Furthermore, these IFRGs are specific as personalized molecular biomarkers to assess the infiltration of immunocytes and the effect following immunotherapy.

Several studies have indicated that the inflammatory status and immune cell infiltration promote the initiation of cancer cells, which inspired us to detect the potency of IFRGs with distinguished expression in various clinicopathologic states [24, 25]. Some previous studies that discovered the differential genes in BCa and non-BCa patients also provided insight into the detection at the genetic level. Cancer cell immunogenicity, the TME and the strength of immune activity were demonstrated to closely affect the response of immunotherapies by targeting specific molecules [26, 27]. Jungels C et al. found that in ARID1A identification, recurrence was strongly related to immunogen mutations [28–30]. In addition, based on three tissue-related and one urine cell-related genomic next-generation sequencing study, large numbers of genes showed the predicted potency on the BCG response. Furthermore, a series of studies have investigated multiple factors predicting the response to anti-PD-1/PD-L1 immunotherapy. Lack of HLA haplotypes, JAK1/JAK2 and B2M gene mutations, and increased expression of some immune checkpoints, including CTLA4, IDO, LAG3, TIM-3, TIGIT and VISA, correlated with therapeutic resistance and susceptibility. [24, 31–33] Although a modest increase in anti-PD-1/PD-L1 drugs has been approved by the FDA, there is still some degree of ambiguity regarding the relationship between the PD-L1 expression level and the therapeutic response rate. Whether some potential possibility can be discovered to obtain predictive values of immunotherapies remains to be uncovered.

Although the current observations suggest that some multifactorial models partly increase the accuracy in predicting the response rate of anti-PD-1/PD-L1 therapies, we believe that more integrated and dynamic models will provide more precise signatures predicting prognosis [34, 35]. Recently, attention has been drawn to the implications of detecting the immune checkpoint level in circulating tumor cells to achieve real-time evaluation; however, more efforts are needed to further establish the prediction model [36–38]. These observations motivated us to focus subsequent studies on developing an appropriate and practical protocol for evaluating the immune states and suggesting clinical prognosis in patients with BCa. We utilized an immune-related risk scoring model. Hugo W et al. declared the importance of innate anti-PD-1 resistance gene signatures in predicting clinical outcomes [39]. Peng W et al. indicated that activation of PI3K signaling resulting from the absence of PTEN could be an immune biomarker foreshadowing resistance outcomes [40].

In the current study, we found that some IFRGs, including high expression of AHNAK and CACYBP, are closely related to advanced grade, staging, T-stage and N-stage. A strong positive correlation was observed between the expression of EDNRA and age, grade, stage, and T-stage. In addition, the increased expression of IGF1 significantly positively affected the grade, stage and T-stage. Less MMP9 expression was negatively correlated with grade and T-stage. The expression of NAMPT and SLIT2 was positively correlated with grade, stage and T-stage. STAT1 is an extraordinary IFRG that is differentially expressed in different grades, stages, T-stages and M-stages. The higher expression of CALR was correlated with advanced grade and early M-stage. The overexpression of RBP7 has a stronger relationship between the higher stage and grade. It was proven that increased expression of PAEP, PPY and RAC3 was closely related to higher grade. Overexpression of SH3BP2 was related to early N-stage.

To further verify the relevance of IFRGs to tumor characteristics and the indicating effects, we analyzed and assessed the expression levels of CACYBP in a non-BCa population and patients with BCa with various T-stages at the genetic and protein levels. More obvious expression was observed in various BCa cell lines than in uroepithelium cells. Moreover, with the more advanced T-stages, the expression level was significantly elevated. The results in BCa tissues were consistent with those *in vitro*.

Although we highlight the roles of some sIFRGs in indicating prognosis and verified the expression level of CACYBP in cell lines and tumor tissues, some limitations remain to be further discussed. First, proteomics and metabolomics should be included in the model, combined with immunogenomics, to achieve a more comprehensive analysis and prediction. Thus, the clinical importance of these IFRG signatures is still poorly investigated and requires further verification. Third, in addition to CACYBP, some other sIFRGs involved in our risk scoring model should also be examined *in vivo* and *in vitro*.

In conclusion, we comprehensively assessed the effects of sIFRGs in the prediction of the immune-related clinical outcomes of BCa. Our results provide novel insight into immunotherapies and establish an appropriate risk scoring model for evaluating prognosis in BCa.

## MATERIALS AND METHODS

### Human bladder cell lines

Bladder cancer tissues and normal adjacent tissues were collected from 50 patients admitted to the First Affiliated Hospital of Chongqing Medical University and diagnosed with bladder cancer. Human normal bladder epithelial cell lines SV-HUC-1 and bladder cancer cell lines (BIU-87, TCC-sup, T24, 5637, and UMUC-3) were purchased from the American Type Culture Collection (Manassas, Virginia, USA). Cells were cultured in 1640 and DMEM supplemented with 10% fetal bovine serum (FBS), 100 U/ml penicillin and 100 mg/ml streptomycin (Gibco, Gaithersburg, MD, USA). Cells were incubated at 37°C in 5% CO2. The medium was changed every 1-2 days.

### Data download and preprocessing

Transcriptome RNA-sequencing data of BLCA samples were downloaded from the TCGA data portal (https://portal.gdc.cancer.gov/), which contained data from 19 nontumor tissues and 411 primary BCa samples. Clinical data about these patients were downloaded and extracted. Raw data were prepared for further analyses. These data were updated on September 17, 2019. RNA-seq results were combined into a matrix file using a merge script in the Perl language (http://www.perl.org/). Next, the Ensembl database (http://asia.ensembl.org/index.html) was used to convert the Ensembl IDs of genes into a matrix of gene symbols. IFRGs were obtained from the Immunology Database and Analysis Portal (ImmPort) database (https://www.immport.org/). ImmPort is a database that can accurately update immunological data in a timely manner. The data from ImmPort are a significant basis for immunological research. More importantly, a list of IFRGs provided by the database can be used for cancer research. These genes were identified as having an important role in the immune process.

### Differential gene analysis

To identify IFRGs participating in the pathogenesis of BCa, the R software limma package (https://bioconductor.org/packages/release/bioc/html/limma.html) was used to screen for differentially expressed genes in cancer and adjacent normal tissues. We present all transcribed differential gene analysis data with the screening value of “FDR < 0.05, log_2_ | FC | >1 and P < 0.05”. The differential IFRGs were all extracted from the differential genes. Functional enrichment analysis was performed through the GO and KEGG pathways to explore the underlying molecular mechanisms of differential genes and differential IFRGs.

### Survival-related IFRGs

Differentially expressed IFRGs associated with clinical outcomes in patients with BCa were identified as sIFRGs. sIFRGs were selected by univariate Cox analysis using R software survival packages. Functional enrichment analysis was also performed on IFRGs that are closely related to overall survival (OS). Because these sIFRGs may have clinical applications, their clinical value is also worthy of systematic exploration. In order to explore the interaction between these genes, a PPI network based on the data was constructed on the STRING online database (https://string-db.org/). PPI networks can show relationships between many interacting genes. The criterion for a core gene is no less than five node degrees. Cytoscape software version 3.7.2 was used to display PPI results. We further explored their control mechanisms. TFs directly control the degree of gene expression. Therefore, it is necessary to explore the relationship between potentially competent TFs and clinically relevant IFRGs. Cistrome Cancer (http://cistrome.org/) is a data source that integrates cancer genomics data from TCGA with more than 23,000 ChIP-seq and chromatin accessibility analyses to provide regulatory links between TFs and transcriptomes. The Cistrome Cancer database contains a total of 318 TFs for experimental and computational cancer biology research. Then, we constructed an interaction network of prognosis-related TFs and sIFRGs.

### Creation of the immunogen-related risk score model (IRRS)

The sIFRGs were submitted for multivariate analysis, while the integrated IFRGs were still used as an independent prognostic indicator to develop the IRRS. The IRRS was based on expression data multiplied by Cox regression coefficients. Patients were divided into low-risk groups and high-risk groups according to the median risk score. The value of IRRS was employed to evaluate various subtypes of patients with BCa. The tumor infiltrating immunocytes were evaluated through the TIMMER database. The level of immune infiltration in patients with BCa was downloaded, and the relationship between IRRS and immune cell infiltration was calculated.

To further investigate the relevance of the sIFRGs and clinicopathological features of BCa, we analyzed the relationship between the IRRS and clinicopathologic characteristics, of which the “TNM staging method” is the most common way to describe the tumor status. The division of “T-stage” BCa was based on the depth and extent of tumor invasion, with a deeper and more extensive invasive status in the more advanced T stages. “N-stage” reflects the lymph node metastasis conditions with more and larger metastatic lymph nodes in the more advanced N stages. “M-stage” is distinguished according to whether the tumor exhibits distant metastasis, and advanced M stages usually represent poor tumor conditions. In addition, “staging” is a comprehensive method combining T-stage, N-stage and M-stage to divide patients with BCa into I, II, III and IV staging.

### Western blot

BCa and urothelial cell lines were lysed with RIPA lysis buffer (Beyotime, Haimen, China) and 1% PMSF (Beyotime) at low temperature for 30 min. The protein concentration was quantified using the BCA kit (Beyotime). An equal amount of protein sample was separated by 12% SDS-PAGE and transferred to a polyvinylidene fluoride membrane (Millipore Sigma). The membrane was blocked with 5% skimmed milk at room temperature for 2 hours and incubated at 4°C overnight with appropriate primary antibodies. Then, the membrane was washed with Tween buffer 3 times, and the secondary antibody was incubated for 1 hour at room temperature. Finally, the protein expression level was detected by the chemiluminescence method.

### Immunohistochemical staining

Paraffin-embedded BCa tissue sections were dewaxed with xylene, followed by rehydration with reduced concentrations of ethanol (100%, 95%, 85%, and 75%). To obtain the antigen, the sections were immersed in sodium nitrate buffer and heated in sodium citrate for 30 minutes. Then, the endogenous peroxidase activity was removed with 3% hydrogen peroxide and blocked with normal goat serum at room temperature for 15 minutes. The sections were incubated with the primary antibody at 4°C overnight. Following rewarming at room temperature, ascorbic acid was applied at 37°C for 30 minutes. Next, streptavidin-horseradish peroxidase conjugate was added at 37°C for 30 minutes, followed by staining with DAB for 1 min and hematoxylin for 10 s. Finally, PROPLUS (IPP, V.6; MydiyByNeNICs, SilverSpring, MD, (US)) was used to analyze the expression levels of various markers. In addition, the sustained intensity and response intensity were regarded as the parameters to quantitatively measure and evaluate the protein expression levels.

### Real-time quantitative PCR

TRIzol (Invitrogen) was used to extract total RNA from BCa and urothelial cell lines under various experimental conditions according to the manufacturer's instructions. The PrimeScript RT kit (Osaka, Japan of TaKaRa) combined with RNA (1 μg) was utilized to reverse transcribe cDNA. The reaction steps were as follows: 37°C for 15 min and 85°C for 5 s. Quantitative polymerase chain reaction (qPCR) was performed on an ABI 7500 real-time PCR system (Applied Biosystems) using the SYBR-Green method (TaKaRa). The reaction cycle conditions were performed (95°C for 30 s, followed by 40 cycles of 95°C for 5 s and 60°C for 34 s); the primer sequences are shown in Table 4. Three replicate assays were performed for each cDNA sample.

**Table 4:** The primer sequences of CACYBP and β-actin.

**CACYBP**	F primer (5’-3’)	GCTGCTGTGGTTGCTCCCAT
	R primer (5’-3’)	TGCACCTGCACATTCTCAGTGG
**β-actin**	F primer (5’-3’)	AAACGTGCTGCTGACCGAG
	R primer (5’-3’)	TAGCACAGCCTGGATAGCAAC

Note: F primer: forward primer; R primer: reverse primer.

### Statistical analysis

Gene function enrichment analysis was based on the R software packages of cluster profiler, org.Hs.eg.db, and enrichplot. The predicted target of the ROC curve is the prognosis of patients with BCa. To verify the prognostic performance, the ROC curve was drawn by the survival ROC package of the R software. The survival time, survival status and risk score of patients with BCa were used to predict the prognosis over a 5-year period. Then, the ROC curve was drawn, and the value of the AUC was calculated. The abscissa is the false positive rate, and the ordinate represents the true positive rate. The AUC generally ranges between 0.5 and 1, and values close to 1 indicate greater accuracy. Variations in clinical parameters were determined using independent t-tests. P<0.05 was considered statistically significant.

### Ethics statement

Informed consent forms were signed by all patients before the study. The research protocol has been approved by the Ethics Committee of The First Affiliated Hospital of Chongqing Medical University and is based on the ethical principles of medical research involving human subjects in the Helsinki Declaration.

## Supplementary Material

Supplementary Figures

Supplementary Table 1
