# Supplementary material for: Establishment of a novel risk score model by comprehensively analyzing the immunogen database of bladder cancer to indicate clinical significance and predict prognosis
Source: Aging (Albany NY). 2020 Jun 22;12(12):11967–89. doi: 10.18632/aging.103364 (PMC7343485; doi:10.18632/aging.103364)
Supplement: Supplementary Figures [file aging-12-103364-s002..pdf]

SUPPLEMENTARY FIGURES

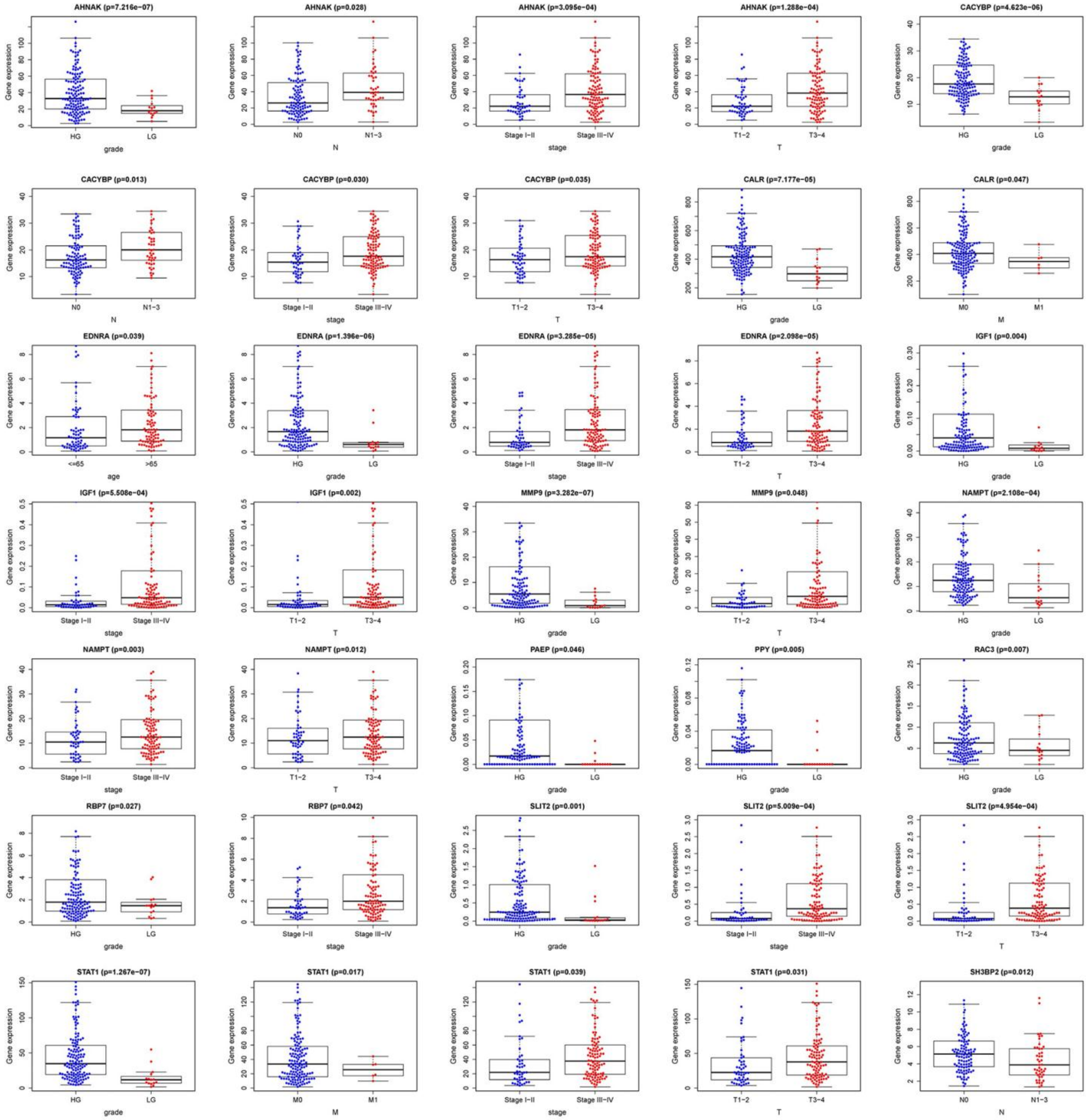

Supplementary Figure 1. The relationships between compositions of IRRS and clinicopathological and demographic characteristics

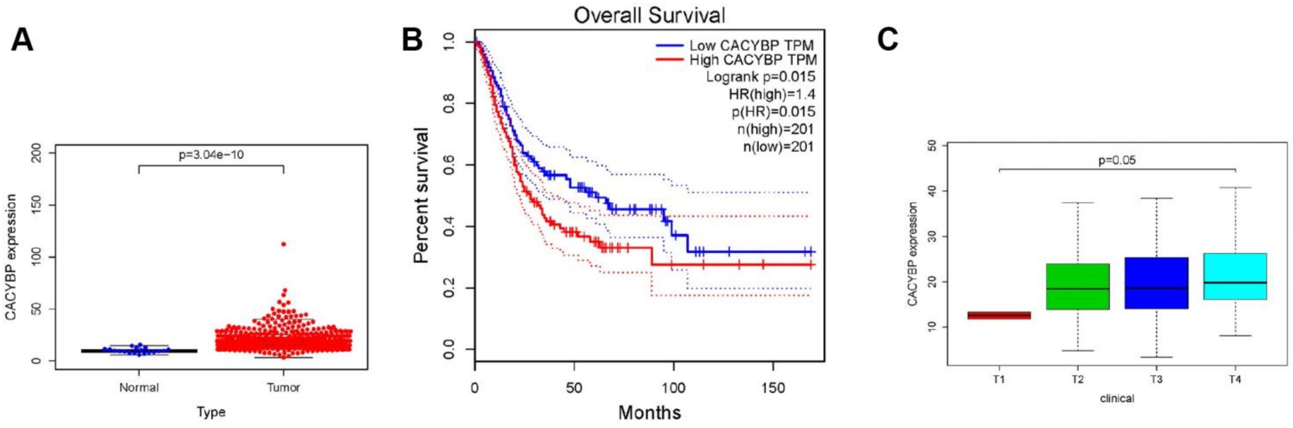

Supplementary Figure 2. The characteristics of CACYBP. CACYBP significantly more expressed in BCa tumor tissues (**A**). The higher expression levels of CACYBP were correlated with poor prognoses (**B**). CACYBP increasingly expressed with the more advanced T-stages (**C**).
