# Supplementary material for: Establishment of a novel risk score model by comprehensively analyzing the immunogen database of bladder cancer to indicate clinical significance and predict prognosis
Source: Aging (Albany NY). 2020 Jun 22;12(12):11967–89. doi: 10.18632/aging.103364 (PMC7343485; doi:10.18632/aging.103364)
Supplement: Supplementary Table 1 [file aging-12-103364-s001..docx]

**Supplementary table: The relevant transcription factors (TFs)**

| **TFs** | **Immune genes** | **Correlation** | **P value** | **Nature** |
| --- | --- | --- | --- | --- |
| ATF3 | MAP3K8 | 0.455574322615112 | 4.80396203069321E-22 | positive |
| EBF1 | RBP7 | 0.665688788644854 | 6.27068784672334E-53 | positive |
| EBF1 | ELN | 0.409532909095488 | 9.92965420898123E-18 | positive |
| EBF1 | ANXA6 | 0.551931714675778 | 1.66056792683037E-33 | positive |
| EBF1 | NFATC1 | 0.423114305106031 | 6.19306525669462E-19 | positive |
| EBF1 | NFATC4 | 0.445414659933452 | 4.91168396728309E-21 | positive |
| EBF1 | SLIT2 | 0.506719670515272 | 1.12999891869249E-27 | positive |
| EBF1 | OGN | 0.42290898856502 | 6.46446514359991E-19 | positive |
| EBF1 | TGFB3 | 0.419327025038816 | 1.35973962630857E-18 | positive |
| EGR2 | NFATC1 | 0.438409821778418 | 2.33280936098932E-20 | positive |
| GATA3 | NRP2 | -0.40233104428306 | 4.11155923160624E-17 | negative |
| GATA6 | THBS1 | 0.530252738491917 | 1.3387497536413E-30 | positive |
| GATA6 | ELN | 0.605689956031509 | 1.03911147730033E-41 | positive |
| GATA6 | ANXA6 | 0.650976366441634 | 6.08856370396242E-50 | positive |
| GATA6 | SLIT2 | 0.495972252226834 | 2.06862861412975E-26 | positive |
| GATA6 | EDNRA | 0.487278302297467 | 2.01766842051843E-25 | positive |
| GATA6 | IGF1 | 0.523774991094246 | 9.01823297638279E-30 | positive |
| GATA6 | OGN | 0.531852059477647 | 8.30623069952672E-31 | positive |
| GATA6 | TGFB3 | 0.58160699219496 | 7.59144665146414E-38 | positive |
| GATA6 | NRP2 | 0.521111904914702 | 1.95236283654778E-29 | positive |
| GATA6 | PTGER3 | 0.425348252396148 | 3.87623100167632E-19 | positive |
| GRHL2 | ANXA6 | -0.450968305445911 | 1.39162864519633E-21 | negative |
| IRF4 | ANXA6 | 0.43816727066378 | 2.46053395566679E-20 | positive |
| IRF4 | NFATC1 | 0.480581819345998 | 1.11608911492411E-24 | positive |
| LHX2 | RAC3 | 0.431076727272418 | 1.14716081576685E-19 | positive |
| LIN9 | CACYBP | 0.448497461815487 | 2.4459121552699E-21 | positive |
| LMNB1 | CACYBP | 0.433777049202083 | 6.40990780548652E-20 | positive |
| MAFF | MAP3K8 | 0.52706598771486 | 3.4393297335972E-30 | positive |
| MEF2C | CXCL12 | 0.44516068146316 | 5.20042408467213E-21 | positive |
| MEF2C | PDGFRA | 0.407856569567827 | 1.38645130290478E-17 | positive |
| MEF2C | ANXA6 | 0.649709126545019 | 1.08161592160011E-49 | positive |
| MEF2C | NFATC1 | 0.559273496871232 | 1.53806666686478E-34 | positive |
| MEF2C | SLIT2 | 0.439595326148109 | 1.79664323668814E-20 | positive |
| MEF2C | EDNRA | 0.400658692727795 | 5.69072732022629E-17 | positive |
| MEF2C | OGN | 0.405320100697998 | 2.28934846242187E-17 | positive |
| MEF2C | PDGFD | 0.43835891308406 | 2.35906405773056E-20 | positive |
| MEF2C | TGFB3 | 0.404491385518773 | 2.6944607159724E-17 | positive |
| MEF2C | TGFBR2 | 0.469180388344817 | 1.88558860932262E-23 | positive |
| MYH11 | ELN | 0.489508663069154 | 1.13182476459767E-25 | positive |
| MYH11 | ANXA6 | 0.563087242001143 | 4.36524130372136E-35 | positive |
| MYH11 | EDNRA | 0.404004081772478 | 2.96475094844294E-17 | positive |
| MYH11 | OGN | 0.458333477355771 | 2.52066834447485E-22 | positive |
| MYH11 | TGFB3 | 0.428229129577843 | 2.10736412511597E-19 | positive |
| MYH11 | NR3C2 | 0.435811496099859 | 4.12011991092197E-20 | positive |
| NCAPG | CACYBP | 0.404927341240524 | 2.47327461202695E-17 | positive |
| NFATC1 | MMP9 | 0.522216643611995 | 1.41830159486531E-29 | positive |
| NFATC1 | PTX3 | 0.43032817927756 | 1.34677330097512E-19 | positive |
| NFATC1 | ANXA6 | 0.547037408556532 | 7.85242868821991E-33 | positive |
| NFATC1 | NFATC1 | 0.999277576856561 | 0 | positive |
| NFATC1 | SLIT2 | 0.447542112523956 | 3.03811337405062E-21 | positive |
| NFATC1 | IGF1 | 0.468324967787007 | 2.32123432896338E-23 | positive |
| NFATC1 | SPP1 | 0.485529242351675 | 3.16560091553268E-25 | positive |
| NFIC | ANXA6 | 0.510320731586889 | 4.16788878732167E-28 | positive |
| NR4A1 | MAP3K8 | 0.450232684909457 | 1.64683126681799E-21 | positive |
| PRDM1 | IFIH1 | 0.406054249337135 | 1.98089465507568E-17 | positive |
| PRDM1 | STAT1 | 0.402575057603273 | 3.92051379485745E-17 | positive |
| SOX17 | THBS1 | 0.463006838087703 | 8.34210758338996E-23 | positive |
| SOX17 | ELN | 0.588192827953752 | 7.17446087894928E-39 | positive |
| SOX17 | PDGFRA | 0.480441832057762 | 1.15625592405196E-24 | positive |
| SOX17 | PTX3 | 0.525484179391365 | 5.47352210988921E-30 | positive |
| SOX17 | ANXA6 | 0.481112752301245 | 9.75881246556349E-25 | positive |
| SOX17 | NFATC1 | 0.574091648370535 | 1.05040768490824E-36 | positive |
| SOX17 | IGF1 | 0.586612387418764 | 1.26995834383232E-38 | positive |
| SOX17 | OGN | 0.55017610313911 | 2.90784461150618E-33 | positive |
| SOX17 | PDGFD | 0.46167960296629 | 1.1439733508035E-22 | positive |
| SRF | ANXA6 | 0.522775919058914 | 1.20589872597979E-29 | positive |
| STAT1 | TAP1 | 0.826916058887624 | 2.6742389461233E-102 | positive |
| STAT1 | TAP2 | 0.775934141354658 | 2.95102502016364E-82 | positive |
| STAT1 | CXCL10 | 0.619420493398948 | 4.59521404526296E-44 | positive |
| STAT1 | IFIH1 | 0.740757260312566 | 2.79077810591849E-71 | positive |
| STAT1 | STAT1 | 0.999625587137882 | 0 | positive |
| STAT1 | BST2 | 0.450910239766555 | 1.4102689350678E-21 | positive |
| TCF21 | ELN | 0.647513885581039 | 2.90785594928892E-49 | positive |
| TCF21 | PDGFRA | 0.499466858438188 | 8.12892583432421E-27 | positive |
| TCF21 | ANXA6 | 0.440113766222302 | 1.60222161763731E-20 | positive |
| TCF21 | OGN | 0.431264835801547 | 1.10176530307298E-19 | positive |
| TCF21 | TGFB3 | 0.402323161659584 | 4.11788093111316E-17 | positive |
| TEAD4 | TAP1 | 0.402409532625536 | 4.04913118790661E-17 | positive |
| TEAD4 | TAP2 | 0.470984070478308 | 1.21414670209647E-23 | positive |
| TTF2 | ZC3HAV1L | 0.41455284022443 | 3.61326039926325E-18 | positive |
| WWTR1 | THBS1 | 0.543136349679278 | 2.66027506764136E-32 | positive |
| WWTR1 | CXCL12 | 0.43254318791007 | 8.36820776382742E-20 | positive |
| WWTR1 | ELN | 0.439026828790894 | 2.03659943421664E-20 | positive |
| WWTR1 | PDGFRA | 0.457404105967835 | 3.13430543313278E-22 | positive |
| WWTR1 | ANXA6 | 0.635306631662341 | 6.14769014068526E-47 | positive |
| WWTR1 | SLIT2 | 0.496541430233164 | 1.77799666322658E-26 | positive |
| WWTR1 | EDNRA | 0.602288018786624 | 3.82319473571486E-41 | positive |
| WWTR1 | OGN | 0.437720420937392 | 2.71412575467085E-20 | positive |
| WWTR1 | TGFB3 | 0.628104616824164 | 1.29448513313255E-45 | positive |
| WWTR1 | NRP2 | 0.606968524240241 | 6.34249920078422E-42 | positive |
| WWTR1 | TGFBR2 | 0.514933459422017 | 1.14151471373092E-28 | positive |
